# Assessing the Performance of Extended Half-Life Coagulation Factor VIII, FC Fusion Protein by Using Chromogenic and One-Stage Assays in Saudi Hemophilia A Patients

**DOI:** 10.1155/2020/8768074

**Published:** 2020-09-09

**Authors:** Tarek M. Owaidah, Hazzaa A. Alzahrani, Nouf S. Al-Numair, Abdulmjeed O. Alnosair, Amelita M. Aguilos, Mahasen Saleh

**Affiliations:** ^1^Departments of Pathology and Laboratory Medicine, King Faisal Specialist Hospital and Research Center, Riyadh, Saudi Arabia; ^2^Departments of Oncology, King Faisal Specialist Hospital and Research Center, Riyadh, Saudi Arabia; ^3^Departments of Genetics, King Faisal Specialist Hospital and Research Center, Riyadh, Saudi Arabia; ^4^Pediatric Hematology, King Faisal Specialist Hospital and Research Center, Riyadh, Saudi Arabia

## Abstract

**Background:**

The one-stage assay is the most common method to measure factor VIII activity (FVIII : C) in hemophilia A patients. The chromogenic assay is another two-stage test involving purified coagulation factors followed by factor Xa-specific chromogenic substrate.

**Aim:**

This study aimed to assess the discrepancy and correlation between the chromogenic and one-stage assays in measuring FVIII : C levels in hemophilia patients receiving Extended Half-Life Elocta® as a recombinant extended half-life coagulation factor.

**Methods:**

We performed a study comparing the measurements of FVIII : C levels by the chromogenic versus the one-stage assays at different drug levels. Data of FVIII : C levels, dosage, and the time interval from administration to measurement were retrieved from the hospital records. The correlation, mean differences, and discrepancy between the two assays were calculated. The linear regression analysis was used to predict the time interval till reaching 1% FVIII : C.

**Results:**

Fourteen patients with 56 samples were included in the study. Of them, 13 patients were receiving Elocta® as a prophylactic, while one was receiving Elocta® on demand. One-third of these samples showed a discrepancy between the chromogenic and one-stage assays. The two assays were well correlated. Mean differences were significant at the individual and the time interval level. The time since the last Elocta® injection could significantly predict FVIII : C levels (*β* = 0.366, *P* < 0.001).

**Conclusion:**

Our findings suggested a significant difference between both methods; the FVIII : C levels measured by the one-stage assay were less than those estimated by the chromogenic assay. However, the measurements of FVIII levels by the two assays were well correlated but discrepant in one-third of the samples. The levels of FVIII : C reach 1% after 5.4 days since the last Elocta® administration.

## 1. Introduction

Hemophilia A is an X-linked bleeding disorder caused by mutations in the genes for factor VIII (FVIII), affecting about one case per 5000 living male births [1–3]. Patients with mild hemophilia suffer from easy bleeding from external orifices and internal organs, especially with trauma or surgery. In moderate cases, patients tend to bleed after minor injuries; incidents of spontaneous bleeding without an apparent cause may occur, while those with severe hemophilia suffer from frequent, excessive, and spontaneous bleeding since birth [1–5]. The diagnosis of hemophilia is usually based on the clinical symptoms and laboratory analysis, confirming the deficiency of one of the coagulation factors. The severity of hemophilia depends on the extent of coagulation factor deficiency [6, 7]. Depending on the ratio of FVIII clotting protein in the blood, hemophilia had been classified to mild when FVIII is 6–49%, moderate when it is 1–5%, and severe if less than 1% [8, 9]. People with hemophilia A bleed longer than others, internally or externally.

Current treatment options for hemophilia A include factor replacement therapy and nonfactor replacement. The standard of care for patients with hemophilia A is prophylactic FVIII concentrate administration as replacement therapy to compensate for FVIII deficiency [10]. However, up to 30% of patients with severe hemophilia A may develop inhibitor antibodies [11, 12]. Therefore, those patients do not respond optimally to FVIII concentrate administration. In such cases, there are many treatment strategies, such as bypassing agents, high-dose clotting factor concentrates, or Immune Tolerance Induction (ITI) therapy [13].

A crucial step in the management of hemophilia A patients is the continuous monitoring of FVIII : C to avoid the severe decrease in FVIII : C, which might lead to spontaneous bleeding and hemorrhage. Therefore, laboratory testing in hemophilia patients is not only crucial for the diagnosis and the assessment of disease severity but also for monitoring and optimizing the treatment [14].

Recently, personalizing FVIII prophylaxis has been proposed to optimize the required treatment dose according to the individual pharmacokinetic response to FVIII [15, 16]. However, other factors as the types of FVIII assay and the FVIII product used still account for variations among patients and the different samples taken from the same patient [17]. The one-stage assay is the most commonly used method to measure FVIII : C. However, the chromogenic assay is a two-stage test that includes purified coagulation factors followed by a factor Xa-specific chromogenic substrate in the last stage [18]. The disadvantages of the one-stage assay include (1) the need for factor-deficient plasma, (2) the poor reproducibility, and (3) the relatively large reagent variability, while the disadvantages of the chromogenic assay include the high cost of the assay and the unfamiliarity in clinical practice [19].

The literature suggests that some discrepancies exist between the chromogenic and the one-stage assay for the measurements of FVIII : C levels in patients with mild and moderate hemophilia A [18, 20, 21]. In this study, we aimed to assess the discrepancy and correlation between the chromogenic and the one-stage assay in the measurement of FVIII : C levels in hemophilia patients who are receiving Elocta® as a recombinant extended half-life coagulation factor product.

## 2. Methods

We followed the “Strengthening the Reporting of Observational Studies in Epidemiology” (STROBE) statement guidelines when reporting this manuscript [22].

### 2.1. Study Design, Setting, and Duration

This is a case study of hemophilia A patients attending hemophilia clinics in King Faisal Specialist Hospital and Research Center during the period from March to July 2018. The local ethics committee approved the study in January 2018 (RAC number: 2180010).

### 2.2. Eligibility Criteria of the Study Population

Study subjects were consecutively selected according to the following criteria: (1) individuals with hemophilia A, (2) all individuals who are on Elocta® as a recombinant extended half-life coagulation factor product, and (3) individuals who have given informed consent. Individuals who developed inhibitor antibodies were excluded.

### 2.3. Laboratory Testing

Samples for measurement of FVIII activity were collected in citrated tube 3.2 for each patient at least two samples at 24 hrs from the dose and just before the next dose. Additional samples at 48, 72, 96, and 120 hrs after dose injection had been collected as part of another study for pharmacokinetics. The two methods used for measurement of FVIII activity were the clotting and chromogenic method. The clotting method uses an immune-depleted or deficient plasma (Sta®ImmunoDefVIII) by Stago Diagnostica STA®-Neoplastine® CI Plus and STA®-Unicalibrator which is intended for the determination of Factor VIII (antihemophilic A factor) activity in the plasma. The assay consists of the measurement of the clotting time in the presence of cephalin and activator of a system in which all the factors are present in excess, supplied by the deficient plasma reagent, except factor VIII which is derived from the sample being tested. On the other hand, the chromogenic method (TriniCHROM™ Factor VIII : C) by TCoag Stago Diagnostica, is designed to quantitatively determine Factor VIII : C in the human plasma, as well as the Factor VIII concentrate. Factor VIII : C is a plasma protein that exists with von Willebrand factor as a complex. After activation through thrombin, Factor VIII : C acts as a cofactor to convert Factor X to Factor Xa in the presence of calcium and phospholipids. The quantity of the generated Factor Xa is determined by means of a specific chromogenic substrate, and it is directly proportional to the Factor VIII : C amount in the sample.

### 2.4. Statistical Analysis

Categorical data were summarized as frequencies and percentages, while continuous data were presented as mean and standard deviation. For comparison of categorical and continuous variables, we used the Chi-square test and Student' *t*-test, respectively. A discrepancy was defined as an FVIII : C ratio between the one-stage to the chromogenic assay of ≤0.6. The Spearman correlation coefficient was calculated to represent the correlation between the two assay measurements. An alpha level below 0.05 was considered for statistical significance. All analyses were performed using SPSS statistical software (version 25, for windows).

## 3. Results

### 3.1. Characteristics of the Study Population

Our study included 14 hemophilia A (11 severe and three moderate) patients with body weight ranged between (25 to 100 kg). Of them, 13 patients received Elocta® as a prophylactic treatment (25–40 U/kg two times weekly), and one patient received Elocta® on demand. One patient received Elocta® once a week, three patients received Elocta® twice a week, and eight patients received Elocta® every five days with one patient missing the frequency. The mean (SD) age of the study population was 23.9 (11.8) years. Our cut-off age for pediatric patients was 14 years; three patients were younger than 14 years (all three patients were at 10 years old), and 11 patients were older than 14 years.

### 3.2. The Discrepancy in FVIII : C between the One-Stage and Chromogenic Assays

Out of the 56 samples collected from 14 patients, 20 samples (35.7%) showed discrepancies (one-stage FVIII : C/Chromogenic FVIII : *C* ≤ 0.6), while 36 samples (64.3%) showed no significant differences. About 16 samples (28.5%) were identical.

### 3.3. The Overall Mean Difference in FVIII between One-Stage vs. Chromogenic Assays

**Figure 1:**
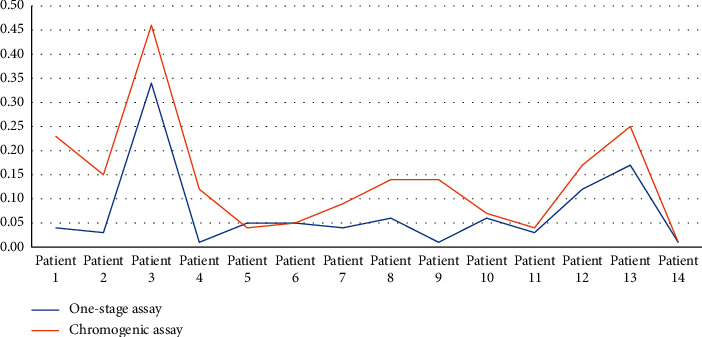
Line chart displaying the mean values of factor VIII levels measured by the one-stage assay and chromogenic assay.

In all samples (*n* = 56), there was a statistically significant difference between the measurement of factor VIII levels by the chromogenic and the one-stage assay using Student' *t*-test (0.14 IU/mL and 0.08 IU/mL; *P* < 0.001). FVIII : C levels by the one-stage assay were lower than those measured by the chromogenic assay. The mean values of factor VIII levels measured by the two assays are shown in Figure 1.

### 3.4. The Difference between the Two Assays Stratified by Time since the Last Elocta® Dose

When the samples were subgrouped according to the time interval since the previous Elocta® dose, the differences between the two assays were statistically significant using Student' *t*-test (Table 1). In all samples, our results demonstrated a significant difference between both assays with a mean difference (mean difference = −0.6, *P* < 0.001).

### 3.5. The Difference between the Two Assays in Individual Patients

When the samples were subgrouped for each subject separately, the differences between the chromogenic and one-stage assays were statistically significant in four out of the 14 patients using Student' *t*-test (Table 2). These differences ranged from 0.002 to 0.19 IU/ml.

### 3.6. The Correlation between the Chromogenic and One-Stage Assays

**Figure 2:**
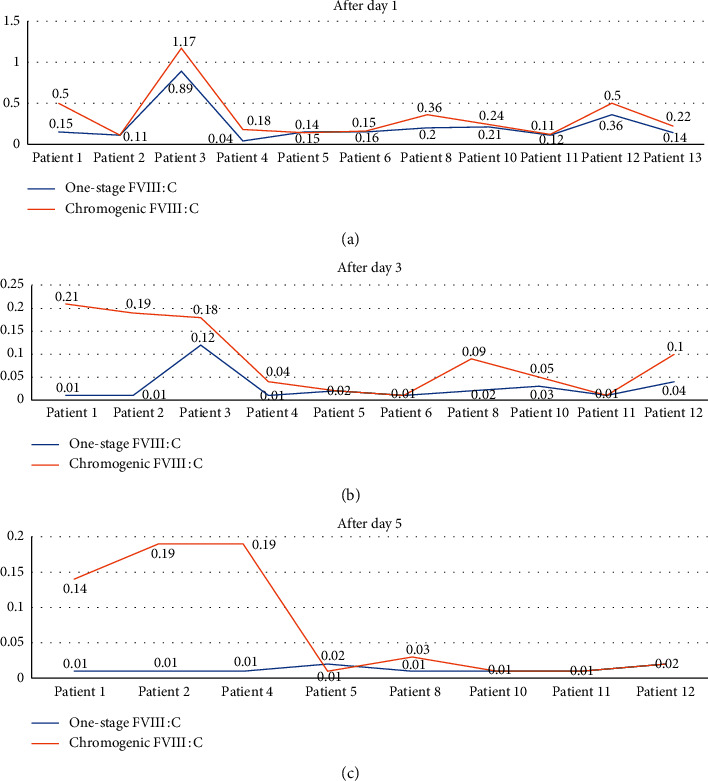
Line charts of the correlation between the chromogenic and one-stage assays' measurements of FVIII levels after day 1, day 3, and day 5.

There was a statistically significant Spearman correlation between the chromogenic and one-stage assays (*r* = 0.918, *P* < 0.001). The correlations between the two measures were statistically significant in 9 out of 12 patients (those who had more than two different samples). The correlation coefficient was positive in all cases except one patient and ranged from 0.3 to 1 with most cases (11/12) having a strong correlation between the two assay measures (Table 3). The line charts of the correlation between the two assays after one day, three days, and five days are shown in Figure 2.

### 3.7. Prediction of Plasma Factor VIII Levels after Injection by Time since the Last Injection

In this part, we took the total samples for each patient at 24, 48, 72, 96, and 120 days. Patients younger than 14 years were excluded. The time since the last injection was calculated and presented as the number of days. A linear regression analysis model was constructed. The linear regression model showed that the time interval since the last injection could significantly predict plasma factor VIII levels measured by the chromogenic assay (*β* = 0.366, *P* < 0.001). The regression equation from this model was Predicted plasma FVIII : *C* = -0.065 Time Interval since the Last Injection + 0.366.

Based on this regression equation, the plasma levels of factor VIII in adult patients with severe-to-moderate hemophilia A decrease to 0.01 IU/mL after 5.4 days since the last injection.

## 4. Discussion

### 4.1. Study Findings and Comparisons

Our study showed that the FVIII : C measured by chromogenic and one-stage assays was discrepant in 35% of the population. Although the FVIII : C levels measured by the one-stage assay were less than those estimated by the chromogenic assay, this difference was statistically significant in some individuals and not significant in others. Furthermore, a statistically significant difference was detected between both assays in all samples (*P* < 0.001). The difference between the chromogenic and the one-stage assay ranged from 0.002 to 0.19 IU/mL. Therefore, there was a strong correlation but not agreement between the chromogenic and the one-stage assay. This disagreement can be attributed to the genetic differences as proposed by Cid et al. [23] and Pavlova et al. [24]. Our regression analysis showed that, in adult patients with moderate-to-severe hemophilia A, the level of FVIII decreases to 0.01 IU/mL after about 5.4 days from the last Elocta® injection, highlighting that the prophylactic dose of Elocta® can be given up to every five days.

### 4.2. Previous Studies

Previous reports in the literature have shown discrepancies between the chromogenic and the one-stage assay in measuring FVIII : C [20]. The literature suggests that the discrepancies between the two assays occur in a substantial proportion of mild and moderate (nonsevere) hemophilia A patients [18, 21].

Cid et al. [23] studied the discrepancy between the chromogenic and the one-stage assays for the measurement of FVIII levels in 163 patients with hemophilia A. They detected discrepancies in 20% of the patients. Moreover, the mutations of FVIII were associated with either higher or lower levels of FVIII by the one-stage assay and normal levels of FVIII with the chromogenic assay, suggesting that the discrepancies might be due to genetic variations among individuals.

Another study by Pavlova et al. [24] showed that about one-third of the patients with nonsevere hemophilia A show discrepancies in FVIII levels when measured by the chromogenic and one-stage assays. Moreover, these discrepancies were attributed to genetic mutations. This finding is consistent with our study, where 35% of the samples showed a discrepancy.

Kihlberg et al. [25] compared the two assays in 44 plasma samples from 35 hemophilia B patients; they found no discrepancies between the two measurements in the patients with severe hemophilia. However, among those with nonsevere hemophilia, 15 samples showed at least a two-fold higher difference between the two assays with the one-stage assay presenting a lower value compared to the chromogenic assay. Moreover, 14 out of 15 of the discrepant samples belonged to individuals with genetic mutations exposing them to factor deficiency.

Potgieter et al. [18] showed that the results of both assays are well-correlated; however, the one-stage assay might be influenced by nonspecific inhibition [18]. The one-stage assay might misestimate FVIII : C levels. Therefore, when used alone, it might lead to some misclassification of patients; the one-stage assay can show normal FVIII : C in hemophilia A patients while diagnosing normal FVIII : C people as hemophiliacs [18].

Moreover, a recent study showed the difference between the two assays with different incubation times. They found that, in the chromogenic assay, FVIII : C levels were higher after incubation for 2 minutes than after 10 minutes, while in the standard coagulation assay, FVIII : C levels were higher at shorter incubation times than at longer incubation times usually used [26].

Some reports suggest that the chromogenic assay should be the routine practice in clinical laboratories as it is probably more suitable than one-stage clotting [19, 20]. While others recommend that both assays should be accessible in clinical laboratories [18], and laboratory scientists and clinicians should learn about the advantages and pitfalls of each assay so that they can select the suitable assays for each case and interpret them accurately [20].

### 4.3. Strength Points

The strong points of our study are as follows: (1) we analyzed the measurement differences in multiple samples from the same patients allowing the exploration of the correlation and discrepancies within the same patient, and (2) samples were taken at different time points, therefore, allowing the examination of the discrepancies at different time intervals since the last Elocta® injection. Nonetheless, our study is limited by the small sample size and the lack of information about the genetic mutations in the study's population which might explain the measurement discrepancies between the two assays as reported in the literature.

### 4.4. Generalizability and Current Knowledge

This study expands the literature by confirming the discrepancies between the chromogenic and the one-stage assays for the measurement of FVIII : C in hemophilia A patients who are taking Elocta®.

## 5. Conclusions

The current findings of our study showed that the FVIII : C levels measured by the one-stage assay were less than those estimated by the chromogenic assay. However, the chromogenic and the one-stage FVIII assays are well correlated in this population. We recommend that, in Hemophilia A patients treated with Elocta®, the prophylactic dose can be given up to five days. Further studies on a larger sample are required to explore the correlation and agreement between both methods.

## Figures and Tables

**Table 1:** The comparison between the one-stage assay versus chromogenic assay for the measurement of factor VIII levels in the study population.

Time of sample collection	*N*	One-stage assay†	Chromogenic assay†	Mean difference	SE	*P* value	95% CI
>1 day	11	0.22 (0.23)	0.33 (0.31)	−0.10	0.03	0.014	−0.18 to −0.27
>2 days	10	0.08 (0.07)	0.13 (0.10)	−0.05	0.018	0.026	−0.09 to −0.007
>3 days	10	0.02 (0.03)	0.09 (0.077)	−0.06	0.02	0.024	−0.11 to −0.01
>4 days	11	0.02 (0.02)	0.07 (0.06)	−0.05	0.018	0.022	−0.09 to −0.008
>5 days	8	0.01 (0.004)	0.07 (0.08)	−0.06	0.03	0.077	−0.13 to −0.008
Others^*∗*^	6	0.1 (0.11)	0.13 (0.16)	−0.03	0.019	0.105	−0.08 to 0.011
All samples	56	0.08 (0.13)	0.14 (0.18)	−0.06	0.08	˂0.001	−0.08 to −0.04

*N* = number of samples evaluated; † data reported in mean (standard deviation); SE = standard error; CI = confidence interval; others^*∗*^: includes samples collected after more than one week.

**Table 2:** The comparison between the one-stage assay versus chromogenic assay for the measurement of factor VIII levels in the individual cases.

Patient ID	Age	*N*	One-stage assay†	Chromogenic assay†	Mean difference	SE	*P* value	95% CI
Patient 1	25	5	0.04 (0.06)	0.23 (0.15)	−0.19	0.04	0.009	−0.31 to −0.08
Patient 2	24	4	0.03 (0.05)	0.15 (0.03)	−0.12	0.04	0.063	−0.25 to 0.012
Patient 3	57	4	0.34 (0.37)	0.46 (0.48)	−0.11	0.05	0.123	−0.29 to 0.05
Patient 4	32	5	0.01 (0.01)	0.12 (0.07)	−0.11	0.03	0.03	−0.20 to −0.17
Patient 5	28	5	0.05 (0.05)	0.04 (0.05)	0.004	0.002	0.178	−0.002 to 0.01
Patient 6	24	4	0.05 (0.06)	0.05 (0.07)	−0.002	0.002	0.391	−0.010 to 0.005
Patient 7	10	1	0.04	0.09	−0.05	NA	NA	NA
Patient 8	30	5	0.06 (0.08)	0.14 (0.13)	−0.076	0.025	0.040	−0.14 to −0.005
Patient 9	26	2	0.01 (0.11)	0.14 (0.14)	−0.045	0.025	0.323	−0.36 to 0.27
Patient 10	34	5	0.06 (0.08)	0.07 (0.09)	−0.008	0.007	0.033	−0.02 to −0.12
Patient 11	36	7	0.03 (0.04)	0.04 (0.04)	−0.005	0.002	0.103	−0.01 to −0.001
Patient 12	29	5	0.12 (0.14)	0.17 (0.19)	−0.052	0.024	0.104	−0.12 to 0.016
Patient 13	10	3	0.17 (0.11)	0.25 (0.14)	−0.083	0.020	0.054	−0.17 to 0.003
Patient 14	10	1	0.01	0.01	0	NA	NA	NA

*N* = number of samples evaluated; † data reported in mean (standard deviation); SE = standard error; CI = confidence interval; NA = not applicable since one sample was available.

**Table 3:** The correlation between the one-stage assay and chromogenic assay for the measurement of factor VIII levels in the study cases.

Patient ID	Age	*N*	Correlation coefficient	*P* value
Patient 1	25	5	0.97	0.004
Patient 2	24	4	−0.802	0.198
Patient 3	57	4	1	<0.001
Patient 4	32	5	0.38	0.523
Patient 5	28	5	0.996	<0.001
Patient 6	24	4	1	<0.001
Patient 7	10	1	NA	NA
Patient 8	30	5	0.993	0.001
Patient 9	26	2	1	<0.001
Patient 10	34	5	0.991	0.001
Patient 11	36	7	0.994	<0.001
Patient 12	29	5	0.994	0.001
Patient 13	10	3	0.999	0.26
Patient 14	10	1	NA	NA

*N* = number of samples evaluated; NA=not applicable since one sample was available.

## Data Availability

Data used to support the findings of this study are included within the supplementary information file (s).
